# Contraceptive Use Trends from the Periodic Health Assessment Among Female Active Duty U.S. Sailors and Marines, 2018–2023

**Published:** 2025-05-01

**Authors:** Brooke K. Rodriguez, Katherine R. Gonzales, Sarah C. Kelsey

**Affiliations:** Battelle Memorial Institute, supporting the Defense Centers for Public Health–Portsmouth, VA; Defense Centers for Public Health–Portsmouth, EpiData Center, Defense Health Agency

This Surveillance Snapshot highlights trends in birth control methods among female active duty U.S. sailors and marines. Birth control methods are self-reported during annual Periodic Health Assessments (PHAs) of active duty U.S. service members. This analysis captures data on birth control use including long-acting reversible contraceptives (LARCs), short-acting reversible contraceptives (SARCs), intrauterine devices (IUDs), implants, barrier methods, emergency contraception, sterilization, fertility awareness, or lack of use among individuals not actively taking steps to prevent pregnancy. These findings offer insights into active duty females' preferences and behaviors beyond clinical data and may inform Defense Health Agency (DHA) policies for enhancing female force readiness.

This analysis examined responses to question 22 on PHA DD Form 3204, versions 1 and 2, for calendar years 2018–2023. ^1^ The population included active duty female sailors and marines, ages 18-52 years. Version 2 of the form was introduced in August 2021 and retained the contraceptive questioning structure of version 1, allowing women to select why they may not be taking steps to prevent pregnancy. Contraceptive variables and free-text responses were used to determine prevalence of LARCs, SARCs, and other pregnancy prevention methods, which included infertility or sterilization (of the service member or partner), condom use, abstinence, fertility awareness methods, and pregnancy or breast-feeding. Alternatively, respondents could select a response indicating that pregnancy prevention was not needed (e.g., same sex partner[s], intention to conceive).

Among all PHAs completed and certified with medical provider signature (n=277,633), only 24.3% (n=67,430) included responses to assessment items on contraceptive methods. Among women with a response indicating at least 1 birth control method during the study period, the **Figure** illustrates the distribution of birth control methods by type. Self-reported SARC and condom use decreased from 34.6% to 18.1%, and 30.1% to 15.5%, respectively, from 2018 to 2023. Self-reported LARC use showed an upward trend, rising from 19.6% in 2018 to 31.0% in 2023. During the study period, the percentage of active duty women reporting same-sex relationships increased from 0.6% to 1.8%, but remained below 2%; abstinence increased from 0.3% to 2.8%, but remained below 3%; infertility increased from 8.0% to 9.0%; cycle tracking and family planning increased from 1.8% to 3.6%. Breastfeeding as a method to prevent pregnancy remained consistently low during the period of analysis, never exceeding 2.8% of responses. PHAs with free-text responses to ‘other’ contraceptive methods (n=1,713), which could not be classified into the categories described, were excluded from the analysis.

**FIGURE.**
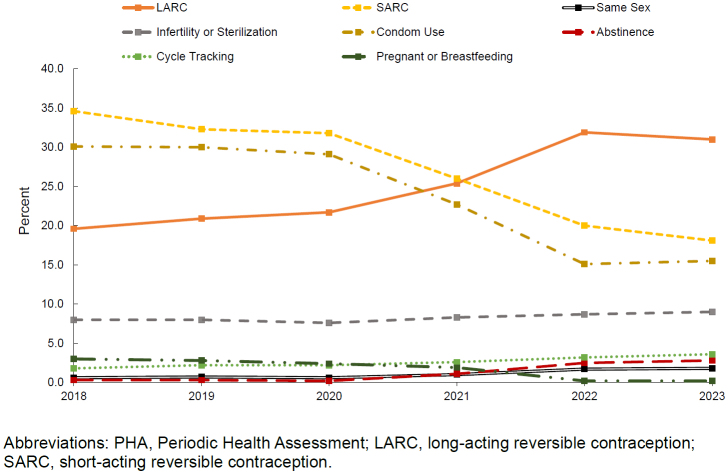
Prevalence of Birth Control Method, Reported on Periodic Health Assessments, Active Duty Sailors and Marines, 2018–2023

It is important to note that PHA data consist of self-reported physical health information and do not represent actual diagnoses, or prescriptions written, filled, or taken. Nevertheless, this information has value, providing insights into reproductive health trends affecting force readiness and resilience, for informed health care strategies.
